# Disseminated Intravascular Coagulation With Purpura Fulminans Presentation of Acute Promyelocytic Leukemia

**DOI:** 10.5811/cpcem.2019.7.43632

**Published:** 2019-10-14

**Authors:** Douglas Ader, Muhammad Durrani, Eric Blazar

**Affiliations:** Inspira Medical Center, Department of Emergency Medicine, Vineland, New Jersey

## Abstract

A 47-year-old male presented to the emergency department with 12 hours of nausea, vomiting, abdominal pain, and a widespread skin eruption with mottled, irregular, purpuric lesions with subsequent rapid decompensation. Laboratory analysis revealed thrombocytopenia, bandemia, elevated metamyelocytes, abnormal coagulation panel, decreased fibrinogen, elevated fibrin split products, renal dysfunction, bacterial rods, dohle bodies, and toxic granulation. Acute promyelocytic leukemia (APML) was confirmed via bone marrow biopsy, flow cytometry, and fluorescence in situ hybridization analysis. Disseminated intravascular coagulation (DIC) may be the initial presentation of APML. When treated promptly, APML can achieve high remission rates; however, conditions such as DIC continue to increase mortality requiring early recognition to improve survival rates.

## CASE PRESENTATION

A 47-year-old male with no past medical history presented with nausea, vomiting, diffuse abdominal pain, and a widespread rash that began 12 hours prior to arrival. Physical examination revealed an ill-appearing male with diffuse abdominal tenderness as well as diffuse purpuric, mottled skin (Image). Vital signs revealed a blood pressure of 93/74 millimeters of mercury, heart rate of 120 beats per minute, respiratory rate of 30 respirations per minute, and a rectal temperature of 103.7 degrees Fahrenheit with pulse oxygenation saturation of 94%. His course necessitated fluid boluses, vasopressors, broad-spectrum antibiotics, steroids, and intubation.

His laboratory findings revealed bandemia, thrombocytopenia, acute kidney injury, elevated coagulation markers, decreased fibrinogen, elevated fibrin split products, elevated lactate dehydrogenase, and a lactic acidosis as shown in the Table, along with an unremarkable computed tomography of his abdomen and pelvis without contrast. The presumptive diagnosis was the development of acute promyelocytic leukemia (APML) with disseminated intravascular coagulation (DIC), an uncommon skin presentation called purpura fulminans (PF), and septic shock.

## DISCUSSION

The patient was transferred to a tertiary care center and underwent bone marrow biopsy, flow cytometry, and fluorescence in situ hybridization analysis confirming APML. APML constitutes 10% of all adult acute myelogenous leukemia and has a high incidence of life-threatening hemorrhage secondary to fibrinolytic-type DIC, which has been attributed to both the development of APML as well as sepsis secondary to APML.^1^,^2^ There is a high misdiagnosis and delayed treatment rate secondary to the varied and complex presentation. APML results from chromosomal abnormalities that involve a balanced translocation between chromosomes 15 and 17 with an incidence of 600–800 cases annually in the United States.^3^ The average age at time of diagnosis is between 20 and 50 years of age.

APML should be considered in those with presentations of bleeding, DIC, or infections with laboratory abnormalities of thrombocytopenia, anemia, and subnormal to elevated white blood cell count. In cases of DIC, scoring algorithms use platelet count, level of fibrin markers such as d-dimer and fibrin split products, coagulation studies, and fibrinogen levels to arrive at a diagnosis. Additionally, with DIC, PF may also occur. PF is a poorly understood entity; it occurs secondary to endovascular thrombotic events and endothelial damage, which contribute to the characteristic purpuric appearance.^4^ The mainstay of treatment is aggressive supportive care as well as identifying and treating the underlying coagulopathy and infections, in addition to induction therapy with all-trans-retinoic-acid and chemotherapeutic agents in consultation with a hematologist.^5^

**CPC-EM Capsule**

What do we already know about this clinical entity?

*Acute promyelocytic leukemia (APML) is associated with a high incidence of fibrinolytic-type disseminated intravascular coagulation (DIC).Treatment is supportive*.

What is the major impact of the image(s)?

*The image reinforces what purpura fulminans looks like and highlights the occurrence of DIC in APML and the importance of early recognition and treatment*.

How might this improve emergency medicine practice?

*Keeping a broad differential with knowledge of this emergent condition will yield earlier coordination of care and treatment of APML, which carries a high misdiagnosis and mortality rate*.

## Figures and Tables

**Image:**
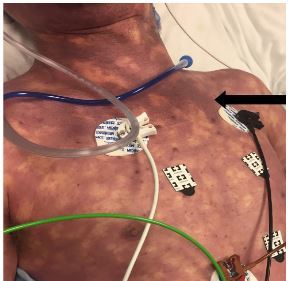
Purpuric mottled skin eruption resulting from disseminated intravascular coagulation (arrow).

**Table:** Abnormal laboratory findings in patient diagnosed with acute promyelocytic leukemia.

Laboratory findings	Values	Reference ranges
White blood cell	9.9×10^3^ × 1000/mm^3^	4–11 × 1000/mm^3^
Hemoglobin	16.1 g/dL	12–18 g/dL
Platelets	27×10^3^ × 1000/mm^3^	150–450 × 1000/mm^3^
Manual differential	26% bands, 6% metamyelocytes, as well as many bacteria rods, Dohle bodies, and evidence of toxic granulation.	Segmented neutrophils: 50%–70%. Band forms: 0–5%. Lymphocytes: 30%–45%. Monocytes: 0–6%. Basophils: 0–1%. Eosinophils: 0–3%
Coagulation panel	Prothrombin time: 24.9 seconds, partial thromboplastin time: 151 seconds, INR: 2.1	Prothrombin time: 11–13 seconds, partial thromboplastin time: 25–35 seconds, INR: 0.8–1.2
Fibrinogen	<80 mg/dL	200–400 mg/dL
Fibrin split products	>40 ug/ml	<10 ug/ml
Creatinine	3.2 mg/dL	0.7–1.3 mg/dL
Lactate dehydrogenase	868 u/L	80–225 u/L
Lactic acid	11.5 mmol/L	0.7–2.1 mmol/L

*mm^3^*, cubic millimeters; *g/dL*, grams per deciliter; *INR*, international normalized ratio; *mg/dL*, milligrams per deciliter; *ug/ml*, micrograms per milliliter; *mmol*, millimoles; *L*, liter; *u*, unit.
